# DNA Methylation Profiling for the Diagnosis and Prognosis of Patients with Nontuberculous *Mycobacterium* Lung Disease

**DOI:** 10.3390/cimb43020038

**Published:** 2021-06-28

**Authors:** Jee Youn Oh, Young Kyung Ko, Jeong-An Gim

**Affiliations:** 1 Division of Pulmonary, Allergy and Critical Care Medicine, Department of Internal Medicine, Korea University Guro Hospital, Seoul 08308, Korea; happymaria0101@hanmail.net (J.Y.O.); youngsoka@naver.com (Y.K.K.); 2 Medical Science Research Center, College of Medicine, Korea University Guro Hospital, Seoul 08308, Korea

**Keywords:** DNA methylation, nontuberculous *Mycobacterium*, tuberculosis

## Abstract

The incidence of nontuberculous *Mycobacterium* (NTM) lung disease is rapidly increasing; however, its diagnosis and prognosis remain unclear while selecting patients who will respond to appropriate treatment. Differences in DNA methylation patterns between NTM patients with good or poor prognosis could provide important therapeutic targets. We used the Illumina MethylationEPIC (850k) DNA methylation microarray to determine the pattern between differentially methylated regions (DMRs) in NTM patients with good or poor prognosis (*n* = 4/group). Moreover, we merged and compared 20 healthy controls from previous Illumina Methylation450k DNA methylation microarray data. We selected and visualized the DMRs in the form of heatmaps, and enriched terms associated with these DMRs were identified by functional annotation with the “pathfinder” package. In total, 461 and 293 DMRs (|Log2 fold change| > 0.1 and *P* < 0.03) were more methylated in patients with four poor and four good prognoses, respectively. Furthermore, 337 and 771 DMRs (|Log2 fold change| > 0.08 and *P* < 0.001) were more methylated in eight NTM patients and 20 healthy controls, respectively. *TGFBr1* was significantly less methylated, whereas *HLA-DR1* and *HLA-DR5* were more methylated in patients with poor prognosis (compared to those with good prognosis). *LRP5*, *E2F1*, and *ADCY3* were the top three less-methylated genes in NTM patients (compared with the controls). The mTOR and Wnt signaling pathway-related genes were less methylated in patients with NTM. Collectively, genes related to Th1- cell differentiation, such as *TGFBr1* and *HLA-DR*, may be used as biomarkers for predicting the treatment response in patients with NTM lung disease.

## 1. Introduction

The incidence and importance of nontuberculous *Mycobacterium* (NTM) lung disease are rapidly increasing [1,2]. NTM diseases are mainly caused by *Mycobacterium avium* complex (MAC), *Mycobacterium kansasii,* and *Mycobacterium abscessus* strains [1]. NTM is prevalent in people with damaged immune systems and requires prolonged treatment; this treatment is accompanied by various side effects such as severe gastrointestinal distress or hearing loss [3]. The prognosis and outcomes of patients with NTM vary based on the genetic and immunological factors [4,5,6]; some cases are associated with spontaneous resolution, whereas others are refractory despite 2–3 years of antibiotic treatment [6,7,8,9]. Therefore, there is an urgent need to develop tools to diagnose NTM at early stages and to identify patients who will respond to appropriate treatment.

DNA methylation involves the addition of a methyl group to the DNA (frequently at the cytosine–guanine dinucleotide (CpG) sites) [10]. DNA methylation profiles may be altered, and they regulate the expression of genes in response to external or internal conditions. Moreover, these profiles are modified during disease progression; thus, they can enable disease identification. Abnormal DNA methylation induced in response to chronic viral infections enables the viruses to evade the host immune surveillance machinery. DNA methylation has also been studied as a biomarker of infection-induced stress [11,12]. The Illumina Infinium MethylationEPIC (EPIC) array enables the simultaneous measurement of the methylation marks at more than 860,000 CpG sites in almost all RefSeq genes [13], thereby enabling the identification of differentially methylated regions (DMRs) between groups. In a previous study, a standard statistical significance for extracting DMR from EPIC chip-derived data was presented; additionally, we tried to extract approximately 1000 DMRs from the two comparison groups [14]. Therefore, in the present study, we used the EPIC array to determine the correlation between DNA methylation profiles and disease progression in NTM. Changes in the DNA methylation patterns of promoters and enhancers regulate gene expression; thus, investigating epigenetic mechanisms at a genome-wide level in humans can bridge the gap between NTM susceptibility and gene expression variation.

An epigenome-wide association study (EWAS) is an additional method used to identify effective epigenetic biomarkers, which can also be used for the identification and prognosis of NTM lung disease. To date, EWAS has been performed to identify altered DNA methylation patterns in several complex diseases, such as diabetes, obesity, schizophrenia, and respiratory diseases [15,16,17,18]. DNA methylation patterns have been studied in *Mycobacterium tuberculosis* infection samples and have been used as biomarkers [19]. Moreover, immune-related factors, used to predict the progression of NTM, have been identified in NTM samples, [20]. Serum from patients with NTM has also been used to detect the association between NTM and other diseases, such as rheumatoid arthritis, bronchiectasis, chronic obstructive pulmonary disease, and cystic fibrosis patients [21]. Nevertheless, to date, no EWAS has been performed for NTM prognosis or to compare the DNA methylation patterns between patients with NTM and healthy controls. Therefore, in the present study, we evaluated the association of the DNA methylation profiles between NTM patients with poor or good prognoses using the EPIC platform, and merged these data with those of a previous DNA methylation study on healthy controls without any respiratory diseases using the Illumina 450k methylation array. Furthermore, we conducted a comprehensive bioinformatic analysis of the DNA methylation patterns between the two data sets and identified and visualized the DMRs. Based on these DMRs, enriched terms, such as T helper (Th)-cell differentiation, mitophagy, spliceosome, adherens junction, diseases, or cancer (e.g., breast cancer, thyroid cancer, and chronic myeloid leukemia), signaling pathways (e.g., Wnt, mTOR, AMPK, notch, and sphingolipid), and cell cycle were selected to depict the upset plots and networks.

## 2. Materials and Methods

### 2.1. Sample Collection

Eight patients with NTM, from the Division of Pulmonology, Department of Internal Medicine, Korea University Guro Hospital, were enrolled in this study. This study was approved by the Institutional Review Board of Korea University Guro Hospital (2017GR0012). The patients agreed to provide blood and clinical data with informed consent. All investigations were conducted in accordance with the principles of the Declaration of Helsinki. The biospecimens and data used for this study were provided by the Biobank of Korea University Guro Hospital, located in Korea.

NTM was diagnosed in accordance with the official ATS/IDSA statement [22]. NTM prognosis was classified as good or bad based on the treatment outcome definitions proposed in an NTM-NET consensus statement [6]. Patients with cured disease were grouped as having a good prognosis, whereas those with treatment failure were classified as having a poor prognosis.

### 2.2. DNA Extraction and Methylation Microarray

DNA was isolated from all eight whole blood samples using the BioRobot EZ1 (Qiagen) system according to the manufacturer’s instructions. We provided 1.0 µg of extracted DNA to Macrogen, Inc. (Seoul, South Korea) for methylation microarray analysis. DNA quality control was confirmed using the Infinium FFPE QC Kit (Illumina, San Diego, CA, USA), and DNA restoration was performed using the Infinium HD FFPE DNA Restore Kit (Illumina). Bisulfite conversion was performed using the EZ-96 DNA Methylation Kit (Zymo Research, Irvine, CA, USA), and a methylation microarray was performed using the Infinium MethylationEPIC BeadChip Kit (Illumina, USA). The iScan system (Illumina) was used to read the BeadChips.

Array data were exported, processed, and analyzed using Illumina GenomeStudio version 2011.1 (Methylation Module version 1.9.0) and R version 4.0.3. Each methylation data point was represented by fluorescent signals from methylated (M) and unmethylated (U) alleles. Thereafter, the ratio of fluorescent signals was computed from two alleles as β = (max(M, 0))/(|U| + |M| + 100). Raw β-values were extracted as 865,918 CpGs. Furthermore, background correlations and dye bias equalization were made using the lumi package in R. Beta-mixture quantile normalization was performed to reduce the assay bias using the BMIQ package in R.

### 2.3. Merged Public Data Set

We used publicly available DNA methylation data from the Illumina 450k methylation array based on samples from 446 people included in the Korean Genome and Epidemiology Study Ansan-Ansung (KoGES-ASAS) [23]. Twenty healthy controls were included in this cohort. Sex, age, and BMI crucially affected the NTM diagnosis and prognosis. Therefore, we matched the healthy controls and patients based on age, sex ratio, and BMI, and selected individuals without any respiratory diseases. We further performed ANCOVA to adjust the age, sex, BMI, and underlying diseases for epigenetic markers. Probes common to the 450k and EPIC datasets were combined using the R “merge” function, and the ComBat method was employed to adjust for the batch effects.

### 2.4. Bioinformatic Analysis and Visualization

To analyze and visualize the characteristics between the two groups, we used the R version 4.0.3 package. Differences between the groups were identified using a *t*-test and visualized using a volcano plot. |Log2 fold change| and *P*-value were defined as the thresholds, which were adjusted according to the DMR patterns between the two groups. The heatmap and hierarchical clustering plot were constructed using the pheatmap package in R. Volcano plots of DMRs were obtained using the plot function in R with Log2 fold change on the x-axis and the transformed –log10 *P*-values on the y-axis. The R package “pathfinder” was used to select terms enriched by the identified DMRs, and these enriched terms were depicted using upset plots and networks with the “Upset_plot” and “term_gene_graph” functions, respectively [24].

## 3. Results

### 3.1. Clinical Characteristics

The clinical characteristics of the study groups are summarized in Table 1. All eight patients with NTM, aged 42–78 years and who underwent treatment, were infected with the same subtype (MAC). The mean age was similar in the two groups (59.1 ± 11.2 in the NTM group and 65.5 ± 5.4 in the healthy control group); moreover, males comprised 62.5% of the patients in the NTM group and 65% in the healthy control group. Patients with NTM exhibited a relatively low body mass index (BMI = 19.7 ± 3.7 kg/m^2^); therefore, we matched these to patients in the healthy control group who had a similar BMI (mean 20.5 ± 1.0 kg/m^2^).

### 3.2. Identification of DMRs According to NTM Prognoses and between Patients and Healthy Controls

**Figure 1:**
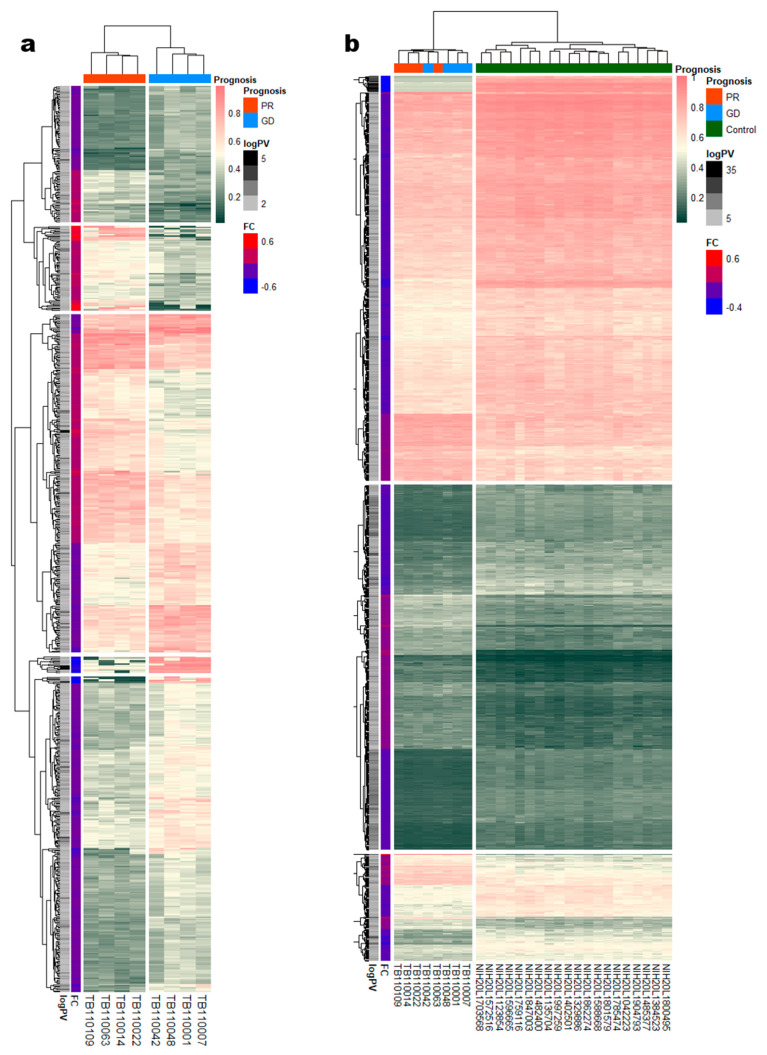
Differential DNA methylation between the NTM prognosis groups and between patients with NTM and healthy controls. (**a**) Heatmap depicting the fold change in methylation between NTM patients with good or poor prognosis. There were 754 DMRs; the poor prognosis group (red row, PR) accounted for 461 more methylated DMRs, and the good prognosis (blue row, GD) group accounted for 293 more methylated DMRs. Unsupervised clustering of eight samples was observed between the prognosis groups. (**b**) Heatmap depicting the fold change between patients with NTM and healthy controls. There were 1108 DMRs; the NTM patient (red column annotation bar, PR; blue column annotation bar, GD) group accounted for 337 more methylated DMRs, and the control (green column annotation bar, GD) group accounted for 771 more methylated DMRs. Unsupervised clustering of 28 samples was observed between patients with NTM and the controls.

**Figure 2:**
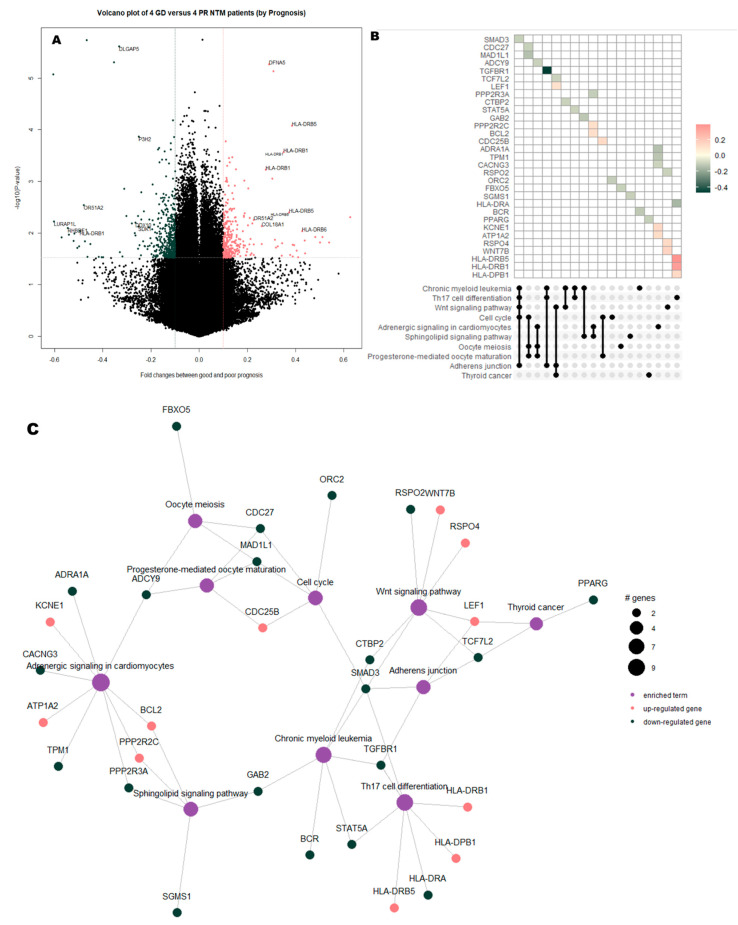
Enriched terms and functional enrichment network of the DMRs between NTM patients with good or poor prognosis. (**a**) Volcano plot illustrating DMRs (defined as a fold change in methylation > 0.1 (red) or < −0.1 (green) with *P*-value < 0.03) in NTM patients with poor prognosis. (**b**) Upset plot indicating the methylation patterns of 55 genes with 10 associated enriched terms. (**c**) Network plot: the color of the circles represents the methylation patterns in patients with NTM, and the size of the enriched circles represents the number of genes involved.

Using |Log2 fold change| > 0.1 and *P*-value < 0.03 as the threshold, we visualized DMRs between NTM patients with good and poor prognoses (*n* = 4 per group) using the “pheatmap” R package. In total, 754 DMRs were identified, including 461 and 293 DMRs that were more methylated in patients with poor and good prognoses, respectively. DMRs are listed according to their β-values and are depicted as a heatmap in Figure 1a; furthermore, all DMRs are plotted as volcano plots in Figure 2a.

**Figure 3:**
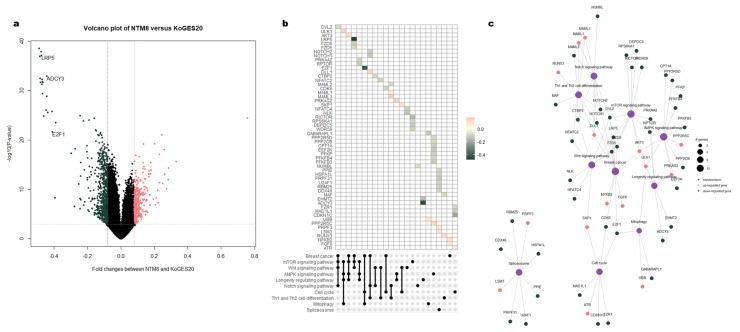
Enriched terms and functional enrichment network of DMRs between eight patients with NTM and the healthy controls. (**A**) Volcano plot of DMRs (defined as a fold change in methylation >0.08 (red) or <−0.08 (green) with *P* < 0.001) in eight patients with NTM. (**B**) Upset plot indicating the methylation patterns of 31 genes and 10 associated enriched terms. (**C**) Network plot: the color of the circles represents the methylation patterns in genes from patients with a poor prognosis, and the size of the circles represents the number of genes involved.

Using |Log2 fold change| > 0.08 and *P*-value < 0.001 as thresholds, we identified 1108 DMRs between patients with NTM and healthy controls, including 337 and 771 DMRs that were more methylated in eight NTM patients and 20 healthy controls, respectively. The DMRs based on the β-values of eight patients with NTM and the batch effect-adjusted β-values of the 20 healthy controls are illustrated in the heatmap in Figure 1b; all DMRs are plotted as volcano plots in Figure 3A.

**Figure 4:**
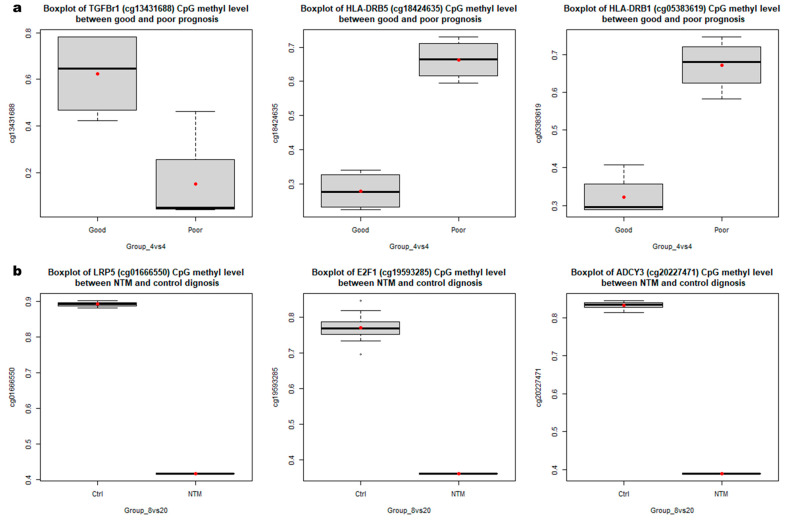
Comparison of the levels of the top three DMRs between (**a**) NTM patients with good and poor prognoses, as well as (**b**) between NTM patients and the healthy controls. * ANCOVA was used to adjust the age, sex, BMI, and underlying diseases for epigenetic markers. All *P*-values were <0.001.

Using these two different DMR analyses, we identified the top three target regions for DMRs between four NTM patients with poor prognosis and four NTM patients with good prognosis (Table S1), as well those for DMRs between eight patients with NTM and 20 healthy controls (Figure 4). Among the top three probes between the two groups of NTM prognosis, two were located on human leukocyte antigen (HLA)-related genes, and all these probes were located in the introns of these genes. Considering the other two loci, the probe was located in the transforming growth factor beta receptor 1 (*TGFBr1*) gene. Among the three probes identified between patients with NTM and healthy controls, two were located in the coding sequences of LDL receptor-related protein 5 (*LRP5*) and E2F transcription factor 1 (*E2F1*). Moreover, the intron in the region of adenylate cyclase 3 (*ADCY3*) revealed a less-methylated pattern in patients with NTM (compared to that in the controls).

### 3.3. Functional Enrichment Analysis of DMRs

We visualized the differences between patients with NTM and healthy controls using volcano plots, upset plots, and functional enrichment networks. Identification of genes with similar methylation patterns and their enriched terms may contribute to the gaining of a better understanding of the etiology of NTM. Therefore, we identified common genes or enriched terms while comparing the diagnosis and prognosis. Two genes, mitotic arrest deficient 1 Like 1 (*MAD1L1*) and C-terminal binding protein 2 (*CTBP2*), were found to be less methylated in two enriched terms: *MAD1L1* was less methylated in the enriched term cell cycle, and *CTBP2* was less methylated in the Wnt signaling pathway. Moreover, other genes or enriched terms differed between the two groups under the two respective conditions.

While comparing NTM patients with good or poor prognosis, it was found that HLA-related genes were more methylated in the patients with poor prognosis (Figure 2b,c), and that these genes were enriched for Th17-cell differentiation. Furthermore, Th17 cell differentiation-related genes, particularly *TGFBr1,* were less methylated in the poor prognosis group. In the network analysis, most of the enriched terms were linked to the less-methylated genes and certain enriched terms, such as Th17-cell differentiation, were linked to more methylated genes in patients with poor prognosis (Figure 2c).

While comparing the eight NTM patients with the 20 healthy controls, *LRP5, E2F1*, and *ADCY3* were all found to be less methylated in the NTM group (Figure 3B). These genes were enriched in breast cancer cells, as well as in the mTOR, AMPK, and Wnt signaling pathways (Figure 3B,C). Most of the enriched terms and genes were linked, except for those in the spliceosome and the eight linked genes. The Notch and AMPK signaling pathways were linked to the more-methylated genes in patients with NTM (Figure 3C).

## 4. Discussion

To the best of our knowledge, this is the first study to evaluate the epigenetic profile of patients with NTM lung disease and to identify novel NTM-related DMRs, which may play pivotal roles in respiratory diseases. The DNA methylation patterns in patients with NTM were clearly distinguishable from those of the healthy controls in terms of the associated genes as well as the enriched terms; moreover, the methylation patterns significantly differed between NTM patients with good or poor prognosis. Presumably, the results and methodology of this study can be used to predict the prognosis of other mycobacterial diseases or to compare it with that in normal subjects. By analyzing the DNA methylation pattern in whole blood, this study provided evidence of a relatively strong prognosis and diagnosis of NTM. The traditional method to detect NTM or TB is time-consuming. The method developed in this study uses a primer to confirm the methylation patterns in many patients. Thus, using this method, NTM or TB can be rapidly and accurately detected (compared to traditional methods); moreover, factors that predict prognosis can also be identified. By confirming the pattern of blood-derived DNA methylation, we can identify disease-related factors underlying the host response to infection.

Numerous identified enriched terms and genes exhibited distinct patterns in NTM patients (compared with the healthy controls) and in NTM patients with poor prognosis (compared with NTM patients with good prognosis). *TGFBr1* and *HLA-DR*—which are related to Th17-cell differentiation—were distinctively methylated in NTM patients with poor prognosis (compared with NTM patients with good prognosis). *LRP5*—associated with mTOR and Wnt signaling—was significantly less methylated in patients with NTM (compared with the controls). Furthermore, genes associated with the AMPK and mTOR pathways and the cell cycle were found to be related to NTM. As several immune-related genes were less methylated in patients with NTM or in those with a poor prognosis, chronic infection and inflammation with NTM might trigger the cellular transformation of host immune cells [25]. Additionally, some DNA methylation patterns may serve as candidate prognostic markers for immune-related respiratory diseases.

Reciprocal regulation between the AMPK and mTOR pathways plays a pivotal role in mycobacterial diseases [26]. The AMPK pathway regulates host autophagy, mitochondrial biogenesis, and metabolic reprogramming, thereby controlling pathological inflammation in mycobacterial infections [27]. Through this pathway, the host defenses are promoted, leading to enhancement of antimicrobial responses against tuberculosis [26]. Hence, NTM lung disease, which is a mycobacterial infection caused by intracellular pathogens, might also be regulated by the AMPK and mTOR pathways.

In addition to the genes associated with the AMPK and mTOR pathways, *MAD1L1* and *CTBP2* were found to be commonly less methylated in patients with NTM (compared with the healthy controls) and in patients with a poor prognosis. *E2F1*—one of the top three DMR probes in NTM—and *MAD1L1* control the inflammatory stimulation of the macrophages and contribute to the activation of T cells in response to the presence of pathogens; these genes are consistently associated with the cell-cycle-enriched term [28]. *CTBP2* is associated with the Wnt signaling pathway and plays a crucial role in mycobacterial disease, as it modulates the inflammatory response and controls the adaptive immune response [29,30,31]. The underlying variations in *CTBP2* expression between patients with TB and healthy controls have been identified in other bioinformatic analyses [32].

A previous pathway analysis revealed differential expression of the microRNAs involved in cell growth, migration, and proliferation, and in the Wnt and TGF-β signaling pathways in NTM lung diseases [33]. The Wnt-pathway-related gene *LRP5* is strongly associated with the progression of pulmonary disease [31], and also regulates the immune mechanisms in TB [30]. This study suggests that the expression of *LRP5* may be increased in patients with NTM, specifically in those with a poor prognosis, and that DNA methylation may play a crucial role in various immune triggers.

*LRP5* is not only involved in the canonical Wnt pathway, but it also regulates the expression of members of the TGF-β gene family [31,34]; TGF-β has been implicated in the pathogenesis of NTM [35]. TGF-β 1 suppresses cytokine-induced macrophage activation [36], which may play a crucial role in regulating the immune response against NTM [37,38]. TGF-β 1 is known to downregulate the expression of HLA-DR in macrophages [37], which is consistent with our results. We found that *TGFB1* was significantly less methylated, whereas HLA-DR genes were significantly more methylated in NTM; both of these genes were associated with Th17-cell differentiation. Th17 cells induce excessive neutrophilic pulmonary inflammation in MAC [39,40]; moreover, the serum concentrations of Th17-related cytokines reflect the treatment outcome in case of NTM lung disease [41]. Treatment failure in patients with NTM may lead to molecular changes in the Th17 differentiation pathway that is known to be associated with inflammation [41]. In the present study, we found that *TGFB1* showed less methylation, and the HLA-DR genes linked with Th17 could act as markers for predicting the treatment outcomes in patients with NTM. Furthermore, genes such as *TGFB1* that exhibit somatic mutations in patients with NTM are involved in breast cancer, and NTM infection may act as a potential risk factor for chronic inflammation and cellular transformation; several patients with NTM ultimately developed breast cancer [42]. This might explain why certain genes related to breast cancer are linked to NTM diagnosis.

This study had two limitations. First, cross-validation was not performed for the DMRs presented in this study. Future experiments using more patients with NTM or TB and cross-validation experiments are required to validate the DMRs or related genes using techniques such as bisulfite sequencing or real-time RT-PCR. Second, NTM patients exhibiting a pattern that differed from the epidemiologically common sex ratio for MAC lung disease were enrolled. MAC lung disease occurs more frequently in women than in men, but its proportion in the present study revealed a different pattern. However, sex differences did not influence the results because epigenetic markers were significant after adjusting for sex. Further studies that take into consideration the sex ratio for MAC lung disease should be carried out. By overcoming the aforementioned limitations in future studies, we look forward to identifying DNA methylation biomarkers that can explain infectious respiratory diseases such as NTM or TB.

## 5. Conclusions

We evaluated the epigenetic profiles for the diagnosis and prognosis of NTM. *TGFBr1* was significantly less methylated, whereas *HLA-DR1* and *HLA-DR5* were more methylated in patients with a poor prognosis (compared to those with a good prognosis). *LRP5*, *E2F1*, and *ADCY3* were the top three less-methylated genes in patients with NTM (compared to the controls). Collectively, *TGFBr1* and *HLA-DR* may be used as potential biomarkers for predicting the treatment response in patients with NTM lung disease.

## Figures and Tables

**Table 1:** Baseline characteristics of patients with NTM and healthy controls.

	NTM *n* = 8	HC *n* = 20
Age	59.1 ± 11.2	65.5 ± 5.4
Sex, male	5 (62.5)	13 (65)
BMI (kg/m^2^)	19.7 ± 3.7	20.5 ± 1.0
Smoker	2 (25)	8 (40)
Alcohol	2 (25)	3 (15)
Comorbidities		
HTN	1 (12.5)	0 (0)
DM	2 (25)	0 (0)
COPD/Asthma	2 (25)	0 (0)
Heart failure	0 (0)	0 (0)

Notes: Data are presented as mean ± standard deviation for continuous variables and number (%) for categorical variables. Abbreviations: BMI, body mass index; COPD, chronic obstructive pulmonary disease; CKD, chronic kidney disease; DM, diabetes mellitus; HTN, hypertension; HC, healthy controls; NTM, nontuberculous *Mycobacterium*.

## Data Availability

All sequencing data sets presented in this study are available upon request from the corresponding author.

## Supplementary Materials

The following are available online at https://www.mdpi.com/article/10.3390/cimb43020038/s1. Table S1: CpGs located in the top 11 differently methylated regions between patients with NTM with a good prognosis and poor prognosis, and the 10 differently methylated regions between patients with NTM and healthy controls.
