# Supplementary material for: DNA Methylation Profiling for the Diagnosis and Prognosis of Patients with Nontuberculous Mycobacterium Lung Disease
Source: Curr Issues Mol Biol. 2021 Jun 28;43(2):501–12. doi: 10.3390/cimb43020038 (PMC8929150; doi:10.3390/cimb43020038)
Supplement: Supplementary file 1 [file cimb-43-00038-s001.zip › cimb-1240759-supplementary.pdf]

Table S1. CpGs located in the top 11 differently methylated regions between patients with NTM with a good prognosis and poor prognosis, and the 10 differently methylated regions between patients with NTM and healthy controls.

Class	Illumina CpG ID	Gene symbol	Gene description	Accession Number	Location	Direction	Genomic region	NTM good prognosis	NTM poor prognosis	p-value	References
NTM prognosis	cg18424635	*HLA-DRB5*	Major histocompatibility complex, class II, DR beta 5	NM_002125	chr6:32490421	-	Intron	0.279±0.049	0.664±0.051	0.0001	[1]
NTM prognosis	cg05383619	*HLA-DRB1*	Major histocompatibility complex, class II, DR beta 1	NM_002124	chr6:32553920	-	Intron	0.322±0.050	0.673±0.060	0.0003	[2]
NTM prognosis	cg24147543	*HLA-DRB1*	Major histocompatibility complex, class II, DR beta 1	NM_002124	chr6:32554481	-	Intron	0.062±0.010	0.339±0.037	0.0006	[3,4]
NTM prognosis	cg23365293	*HLA-DRB5*	Major histocompatibility complex, class II, DR beta 5	NM_002125	chr6:32489984	-	Intron	0.370±0.037	0.743±0.098	0.0040	[5]
NTM prognosis	cg09949906	*HLA-DRB1*	Major histocompatibility complex, class II, DR beta 1	NM_002124	chr6:32552350	-	Intron	0.832±0.021	0.336±0.146	0.0090	[5-7]
NTM prognosis	cg09465394	*MAML3*	Mastermind like transcriptional coactivator 3	NM_018717	chr4:140764922	-	Intron	0.298±0.040	0.178±0.015	0.0093	
NTM prognosis	cg16699635	*HLA-DRB5*	Major histocompatibility complex, class II, DR beta 5	NM_002125	chr6:32498677	-	Intron	0.392±0.038	0.498±0.030	0.0098	
NTM prognosis	cg13431688	*TGFBr1*	Transforming growth factor beta receptor 1	NM_004612	chr9:101869277	+	Intron	0.625±0.160	0.151±0.180	0.0146	[8,9]
NTM prognosis	cg00579921	*HLA-DRB5*	Major histocompatibility complex, class II, DR beta 5	NM_002125	chr6:32489991	-	Intron	0.530±0.106	0.789±0.075	0.0162	
NTM prognosis	cg21698879	*HLA-DRB5*	Major histocompatibility complex, class II, DR beta 5	NM_002125	chr6:32496326	-	Intron	0.795±0.069	0.618±0.021	0.0171	
NTM prognosis	cg15708909	*HLA-DRB5*	Major histocompatibility complex, class II, DR beta 5	NM_002125	chr6:32487314	-	Intron	0.741±0.107	0.500±0.095	0.0271	

NTM vs HC	cg01666550	*LRP5*	LDL receptor related protein 5	NM_002335	chr11:68183850	+	CDS	0.416±0.0002	0.891±0.006	7.31E-38	
NTM vs HC	cg20227471	*ADCY3*	Adenylate cyclase 3	NM_004036	chr2:25065550	-	Intron	0.388±0.0001	0.833±0.009	1.09E-33	
NTM vs HC	cg19593285	*E2F1*	E2F transcription factor 1	NM_005225	chr20:32267661	-	CDS	0.361 ± 0.00003	0.772±0.033	2.09E-22	[10,11]
NTM vs HC	cg08206623	*CDKN1C*	Cyclin dependent kinase inhibitor 1C	NM_000076	chr11:2907334	-	Upstream1k	0.197±0.007	0.400±0.030	1.40E-19	[12-14]
NTM vs HC	cg14497545	*MAML3*	Mastermind like transcriptional coactivator 3	NM_018717	chr4:140754475	-	Intron	0.665±0.012	0.485±0.044	1.08E-14	[12,15-17]
NTM vs HC	cg07199894	*ULK1*	Unc-51 like autophagy activating kinase 1	NM_003565	chr12:132379104	+	Upstream1k	0.309±0.012	0.168±0.042	7.04E-13	[18,19]
NTM vs HC	cg13810766	*PRKAG2*	Protein kinase AMP-activated non-catalytic subunit gamma 2	NM_016203	chr7:151542452	-	Intron	0.582±0.025	0.422±0.035	1.69E-10	[12,20]
NTM vs HC	cg15321108	*PPP2R5C*	Protein phosphatase 2 regulatory subunit B'gamma	NM_001161725	chr14:102227943	+	Intron	0.263±0.020	0.114±0.020	6.56E-10	
NTM vs HC	cg18440777	*LRP5*	LDL receptor related protein 5	NM_002335	chr11:68157499	+	CDS	0.690±0.021	0.779±0.028	9.09E-08	
NTM vs HC	cg09990596	*PPP2R5C*	Protein phosphatase 2 regulatory subunit B'gamma	NM_001161725	chr14:102227939	+	Intron	0.201±0.022	0.115±0.023	7.61E-07	

Abbreviations: NTM, nontuberculous mycobacterium; HC, healthy control; CDS, coding sequence

1. Lou, J.; Wang, Y.; Chen, J.; Ju, L.; Yu, M.; Jiang, Z.; Feng, L.; Jin, L.; Zhang, X. Effects of soluble and particulate Cr (VI) on genome-wide DNA methylation in human B lymphoblastoid cells. *Mutation Research/Genetic Toxicology and Environmental Mutagenesis* **2015**, *792*, 12-18.
2. Wang, C.-M.; Chang, C.-B.; Lee, S.-P.; Chan, M.W.; Wu, S.-F. Differential DNA methylation profiles of peripheral blood mononuclear cells in allergic asthmatic children following dust mite immunotherapy. *Journal of Microbiology, Immunology and Infection* **2020**, *53*, 986-995.
3. Maltby, V.E.; Lea, R.A.; Graves, M.C.; Sanders, K.A.; Benton, M.C.; Tajouri, L.; Scott, R.J.; Lechner-Scott, J. Genome-wide DNA methylation changes in CD19+ B cells from relapsing-remitting multiple sclerosis patients. *Scientific reports* **2018**, *8*, 1-10.
4. Roudbar, M.A.; Mohammadabadi, M.R.; Mehrgardi, A.A.; Abdollahi-Arpanahi, R.; Momen, M.; Morota, G.; Lopes, F.B.; Gianola, D.; Rosa, G.J. Integration of single nucleotide variants and whole-genome DNA methylation profiles for classification of rheumatoid arthritis cases from controls. *Heredity* **2020**, *124*, 658-674.
5. Shin, J.; Bourdon, C.; Bernard, M.; Wilson, M.D.; Reischl, E.; Waldenberger, M.; Ruggeri, B.; Schumann, G.; Desrivieres, S.; Leemans, A. Layered genetic control of DNA methylation and gene expression: a locus of multiple sclerosis in healthy individuals. *Human molecular genetics* **2015**, *24*, 5733-5745.
6. Kular, L.; Liu, Y.; Ruhrmann, S.; Zheleznyakova, G.; Marabita, F.; Gomez-Cabrero, D.; James, T.; Ewing, E.; Lindén, M.; Górnikiewicz, B. DNA methylation as a mediator of HLA-DRB1* 15: 01 and a protective variant in multiple sclerosis. *Nature communications* **2018**, *9*, 1-15.
7. Miller, S.; Tsou, P.-S.; Coit, P.; Gensterblum-Miller, E.; Renauer, P.; Rohraff, D.M.; Kilian, N.C.; Schonfeld, M.; Sawalha, A.H. Hypomethylation of STAT1 and HLA-DRB1 is associated with type-I interferon-dependent HLA-DRB1 expression in lupus CD8+ T cells. *Annals of the rheumatic diseases* **2019**, *78*, 519-528.
8. de Araújo, É.S.S.; Marchi, F.A.; Rodrigues, T.C.; Vieira, H.C.; Kuasne, H.; Achatz, M.I.W.; Moredo, L.F.; de Sá, B.C.S.; Duprat, J.P.; Brentani, H.P. Genome-wide DNA methylation profile of leukocytes from melanoma patients with and without CDKN2A mutations. *Experimental and molecular pathology* **2014**, *97*, 425-432.
9. Zeng, Z.; Huo, X.; Zhang, Y.; Hylkema, M.N.; Wu, Y.; Xu, X. Differential DNA methylation in newborns with maternal exposure to heavy metals from an e-waste recycling area. *Environmental research* **2019**, *171*, 536-545.
10. Prince, C.; Hammerton, G.; Taylor, A.E.; Anderson, E.L.; Timpson, N.J.; Davey Smith, G.; Munafò, M.R.; Relton, C.L.; Richmond, R.C. Investigating the impact of cigarette smoking behaviours on DNA methylation patterns in adolescence. *Human molecular genetics* **2019**, *28*, 155-165.
11. Silva, C.P.; Kamens, H.M. Experimental and Clinical Psychopharmacology. **2020**.
12. Denham, J.; O'Brien, B.J.; Marques, F.Z.; Charchar, F.J. Changes in the leukocyte methylome and its effect on cardiovascular-related genes after exercise. *Journal of Applied Physiology* **2015**.
13. Ge, Y.; Wu, Q.; Ma, G.; Shao, W.; Liu, H.; Zhang, Q.; Xin, J.; Xue, Y.; Du, M.; Zhao, Q. Hypermethylation of EIF4E promoter is associated with early onset of gastric cancer. *Carcinogenesis* **2018**, *39*, 66-71.
14. Ren, J.; Zhang, B.; Wei, D.; Zhang, Z. Identification of Methylated Gene Biomarkers in Patients with Alzheimer's Disease Based on Machine Learning. *BioMed research international* **2020**, *2020*.
15. Hyun, J.; Jung, Y. DNA Methylation in Nonalcoholic Fatty Liver Disease. *International Journal of Molecular Sciences* **2020**, *21*, 8138.

16. Kuramoto, J.; Arai, E.; Tian, Y.; Funahashi, N.; Hiramoto, M.; Nammo, T.; Nozaki, Y.; Takahashi, Y.; Ito, N.; Shibuya, A. Genome-wide DNA methylation analysis during non-alcoholic steatohepatitis-related multistage hepatocarcinogenesis: comparison with hepatitis virus-related carcinogenesis. *Carcinogenesis* **2017**, *38*, 261-270.
17. Ramos-Lopez, O.; Samblas, M.; Milagro, F.I.; Zulet, M.A.; Mansego, M.L.; Riezu-Boj, J.I.; Martinez, J.A. Association of low dietary folate intake with lower CAMKK2 gene methylation, adiposity, and insulin resistance in obese subjects. *Nutrition Research* **2018**, *50*, 53-62.
18. Rojas, D.; Rager, J.E.; Smeester, L.; Bailey, K.A.; Drobná, Z.; Rubio-Andrade, M.; Stýblo, M.; García-Vargas, G.; Fry, R.C. Prenatal arsenic exposure and the epigenome: identifying sites of 5-methylcytosine alterations that predict functional changes in gene expression in newborn cord blood and subsequent birth outcomes. *Toxicological sciences* **2015**, *143*, 97-106.
19. Salas-Pérez, F.; Ramos-Lopez, O.; Mansego, M.L.; Milagro, F.I.; Santos, J.L.; Riezu-Boj, J.I.; Martínez, J.A. DNA methylation in genes of longevity-regulating pathways: association with obesity and metabolic complications. *Aging (Albany NY)* **2019**, *11*, 1874.
20. Lee, S.-W.; Hwang, H.-H.; Hsu, P.W.-C.; Chuang, T.-Y.; Liu, C.-W.; Wu, L.S.-H. Whole-genome methylation profiling from PBMCs in acute-exacerbation COPD patients with good and poor responses to corticosteroid treatment. *Genomics* **2019**, *111*, 1381-1386.
